# A Novel Unsupervised Method to Identify Genes Important in the Anti-viral Response: Application to Interferon/Ribavirin in Hepatitis C Patients

**DOI:** 10.1371/journal.pone.0000584

**Published:** 2007-07-04

**Authors:** Leonid I. Brodsky, Abdus S. Wahed, Jia Li, John E. Tavis, Takuma Tsukahara, Milton W. Taylor

**Affiliations:** 1 Institute of Evolution, University of Haifa, Haifa, Israel; 2 Department of Biology, Indiana University, Bloomington, Indiana, United States of America; 3 Epidemiology Data Center, Graduate School of Public Health, University of Pittsburgh, Pittsburgh, Pennsylvania, United States of America; 4 Molecular Microbiology and Immunology, St. Louis University, St Louis, Missouri, United States of America; National Institute on Aging, United States of America

## Abstract

**Background:**

Treating hepatitis C with interferon/ribavirin results in a varied response in terms of decrease in viral titer and ultimate outcome. Marked responders have a sharp decline in viral titer within a few days of treatment initiation, whereas in other patients there is no effect on the virus (poor responders). Previous studies have shown that combination therapy modifies expression of hundreds of genes in vitro and in vivo. However, identifying which, if any, of these genes have a role in viral clearance remains challenging.

**Aims:**

The goal of this paper is to link viral levels with gene expression and thereby identify genes that may be responsible for early decrease in viral titer.

**Methods:**

Microarrays were performed on RNA isolated from PBMC of patients undergoing interferon/ribavirin therapy. Samples were collected at pre-treatment (day 0), and 1, 2, 7, 14 and 28 days after initiating treatment. A novel method was applied to identify genes that are linked to a decrease in viral titer during interferon/ribavirin treatment. The method uses the relationship between inter-patient gene expression based proximities and inter-patient viral titer based proximities to define the association between microarray gene expression measurements of each gene and viral-titer measurements.

**Results:**

We detected 36 unique genes whose expressions provide a clustering of patients that resembles viral titer based clustering of patients. These genes include IRF7, MX1, OASL and OAS2, viperin and many ISG's of unknown function.

**Conclusion:**

The genes identified by this method appear to play a major role in the reduction of hepatitis C virus during the early phase of treatment. The method has broad utility and can be used to analyze response to any group of factors influencing biological outcome such as antiviral drugs or anti-cancer agents where microarray data are available.

## Introduction

Treating with peginterferon/ribavirin combination therapy patients who have chronic hepatitis C virus (HCV) infection results in a varied response in terms of outcome and decrease in viral titer [1]–[4]. For patients who respond well there is a sharp decrease in viral titer within 24–48 hours after treatment initiation whereas in other patients there is little or no effect on the viral titer and only temporary, or no, clearance of the virus over a long period [5], [6]. Previous in vitro studies have shown that combination interferon treatment induces or decreases expression of hundreds of genes [7]–[10]. One of the major problems, however, is to identify which of these genes are linked to viral clearance in vivo.

In this paper we report a novel mathematical method to explore the association between decrease in viral titer and changes in gene expression in hepatitis C patients following combination treatment with pegylated interferon and ribavirin. The viral clearance time course profile will not necessarily directly correlate with the gene expression time course profile even if the gene is an active participant of the interferon treatment response because the decrease of the viral levels depends on the interplay of many genes and gene products. Therefore, an indirect approach was used in which the relationship between gene expression across days and viral decrease was examined using inter-patient distances (proximity) according to both characteristics.

Using this approach we selected thirty seven gene probes that were linked with the anti-HCV response during the first 28 days of treatment. A visual demonstration of the association of detected genes with the viral decrease is demonstrated by a comparison of patient clusterings. Indeed, the inter-patient proximities according to the pattern of decrease in virus titer provide an unsupervised clustering of patients based on changes in viral levels. Similarly, the inter-patient proximities according to expressions of the specified genes across time provide another unsupervised clustering of patients. A visual inspection of viral-titer based and selected genes expression based clusterings of patients indicates their close relationship. Since the unsupervised clustering of patients according to the pattern of viral clearance is in good correspondence with an a priori biological categorization of patients into marked, slow and poor response at day 28 [11],the selected subset of genes can be considered as genes that are involved specifically in treatment response. Such genes products may have anti-viral activity or be related to the immune response against virus-infected cells.

## Materials and Methods

### Patient population

The Virahep-C study included a cohort of 401 participants who provided written consent at 8 U.S. clinical centers between September 9, 2002 and January 7, 2004. Per protocol, all participants were treated for up to 48 weeks with pegylated interferon alfa-2a (PEGASYS, Roche Inc. Nutley, NJ) at 180 mcg weekly by self-administered subcutaneous injection and ribavirin (COPEGUS, Roche, Inc. Nutley N.J.) by mouth at 1000 mg/day for those who weighed less than 75 kg or 1200 mg/day for those who weighed at least 75 kg. Treatment was discontinued at week 24 in participants who had a detectable serum HCV RNA virus in duplicate qualitative assays using the Roche Cobas Amplicor HCV Test, v2.0 (sensitivity 50 IU/ml). The primary endpoint of the study was the sustained virologic response, defined as an undetectable serum HCV RNA at week 72 (at least 24 weeks after completion of treatment). The clinical trial was #-NCT00038974.

Fifty two patients for whom gene expression and viral level data were available at all time points were selected for this analysis. Based on the log-decline in serum HCV RNA on day 28 of treatment relative to day 0, as measured by the quantitative Roche Cobas Monitor HCV Test, v2.0 (sensitivity 600 IU/ml) these patients were divided into three groups [11] Patients were identified as poor responders if they had less than a 1.4 log_10_ IU/ml decrease in serum HCV RNA between day 0 and day 28, as marked responders if they experienced more than a 3.5 log_10_ IU/ml decrease or if their viral titers dropped to undetectable by day 28, and as intermediate responders if the log_10_ drop in HCV RNA was between 1.4 and 3.5 IU/ml. For the purpose of unsupervised clustering of viral titers, patients with undetected viral levels by the quantitative assay were assigned a viral level of 599 IU/ml and those whose viral levels were undetected by the qualitative assay were assigned a viral level of 49 IU/ml

This study met all necessary approvals of Institutional Review Boards of each institution participating in the Virahep-C consortium (Beth Isreal Deaconess Medical Center, New York Presbyterian Medical Center, Rush University, University of California at San Francisco, University of Maryland, University of Miami, University of Michigan, and University of North Carolina).

### Cell preparation and RNA extraction

Peripheral Blood Mononuclear Cells (PBMC) were collected in sodium heparin-CPT tubes at day 0, 1, 2, 7, 14 and 28. Samples were shipped overnight from each clinical center to a central repository by express courier at 4°C. Whole blood was diluted with an equal volume (8 ml) of phosphate buffered saline, carefully layered over a 10 ml Ficoll-Hypaque gradient (Amersham/Pharmacia) and centrifuged at 800 rpm for 20 minutes at room temperature. The buffy coat layer was transferred to a 15 ml RNAse-free tube and further diluted with PBS. Tubes were centrifuged at 100-× g for 15 minutes at room temperature. The supernatants were discarded and the PBMC were retained.

The isolation of RNA, quality control, the labeling and hybridization on to the micro-arrays have been previously described [7], [10].

### Array Analysis and Data Processing:

The microarrays were scanned using a dedicated Model 3000 scanner controlled by Affymetrix Microarray Suite 5 software (MAS5). The average intensity on each array was normalized by global scaling to a target intensity of 1000. Data were exported from MAS5 into a custom-designed database (MicroArray Data Portal) in the Center for Medical Genomics (IUPUI, Indianapolis). The data from the microarrays has been deposited with NCBI, GEODATA# GSF7123

### Visualization of patient grouping

Visualizing the patient data was simplified through Principal Component Analysis (PCA) [12] which reduces the dimensionality of the data into a small number of independent components. An alternative method to present data grouping is hierarchical clustering. We used the hierarchical clustering (UPGMA) [13] algorithm for the clustering.

Both PCA and the clustering of patient's groupings according to either viral titer or gene expression data were performed to visualize the correspondence between predefined grouping of patients (marked, intermediate, and poor, highlighted by color) and either gene expression or viral titer based grouping of patients.

### Viral titer based proximity of patients and “candidate” genes

Detection of genes that provided clustering similar to viral titer based clustering of patients was performed by a mathematical method similar to the mirror tree method for inferring protein interactions from phylogenetic distance matrices [14], [15]. This method was applied to gene expression and linked the gene-specific variability of patients with their viral titer variability. The link is a correlation between the matrix of inter-patient distances that is based on viral titer and the individual gene expression based matrix. If a gene expression based matrix correlates with a viral titer matrix, then it can be concluded that there is a link between expression of that specific gene and the viral levels in patients. Such a link considers both the direct correlation between the expression profile of a gene across patients and the viral titer pattern of patients, and the indirect involvement of the gene in the viral level change process. A detailed description of the method follows.

The distance between patients' viral titer changes from day 0 was used to estimate the inter-patient proximity with respect to viral change. Similarly, the gene expression based inter-patient proximity was measured for each gene. The correlations between each gene based matrix proximity and viral titer based matrix-proximity were estimated and genes with the highest correlations were selected.

As metrics of inter-patient proximity two measures were used: Euclidean distance and the coefficient of covariation. The larger the coefficient of covariation, the closer the patients are whereas the smaller the Euclidean distance the closer the patients are. Thus, the inter-patient distances based on viral titer time-course measurements created a grouping of patients that reflected the response of patients to antiviral treatment. On the other hand, the inter-patient proximities based on gene expression measurements across days (gene expression profile) for a specific gene reflected the variability of patients either according to response of this gene to treatment, or according to genetic heterogeneity of patients, or both.

The Euclidean distance between the natural logarithms (ln) of changes in viral levels from baseline (day 0) for patient *i* and patient *j* was defined as:
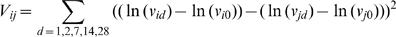
where *v_id_* is the viral titer value for *i* ^th^ patient on day *d*. Denote 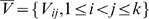 as the vector of Euclidean distances between each pair of patients with dimension *k*(*k*−1)/2, where *k* is the number of patients.

Similarly, the inter-patient Euclidean proximities with respect to the natural logarithm (ln) of the gene expression for the *r* ^th^ gene on day *d* were estimated by
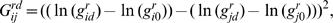
where *g^r^_id_* is the expression of r^th^ gene for *i* ^th^ patient on day *d*. Let 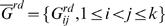 denote the vector of inter-patient proximity according to expression of r^th^ gene at day d. Corresponding to *V_ij_*, define 
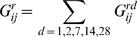
 to be the inter-patient Euclidean proximities with respect to the gene expression for the *r* ^th^ gene. Denote vectors 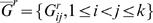 with dimension *k*(*k*−1)/2.

The inter-patient proximities according to expression of all genes was then estimated by the vector of the same *k*(*k*−1)/2 dimension 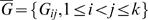, where 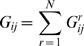, where *N* is the number of genes on the array. Note that 
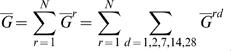


We will refer to the vectors *G̅* *^rd^* and *G̅* *^r^* as GDeu and Geu vectors respectively. Vector *G̅* will be referred to as the main vector (MVeu) of the method and has equal biological importance to the virus titer related vector *V̅* (VTeu) . Indeed, MVeu is the vector of distances between each pair of patients across all genes and all days. These distances reflect differences between patients both due to individual variability and due to the response of patients to the combination treatment. Thus, inter-patient distances across all genes and all days are the superimposition of patient's individual and response differences. The norm of MV is a cumulative measure of how patients are divergent according to their gene expression. Genes that are not co-regulated (the majority of them) have random distances with other genes thus making a minimal impact in the length of the MV. Indeed, gene vectors G with random signals are distributed symmetrically in *k*(*k*−1)/2 space. Under summation they will compensate each other, reducing the norm of the MV vector. Therefore, a large norm for MV is defined by a relatively small number of co-regulated groups of genes, and these groups are related to the biological divergence of patients (including the divergence of their response). From a biological point of view it is important to understand whether the input of genes in MV is correlated with the virus titer vector VT in *k*(*k*−1)/2 space. If they are, then the variability of patients according to gene expression is closely related to their treatment response; if not, then gene expression variability of patients is defined by their other characteristics.

Similarly to Euclidean distance based vectors *G̅* *^rd^*, *G̅* *^r^*, *G̅* and *V̅* of the *k*(*k*−1)/2 space, the covariance based vectors *G̅* *^rd^* ^,cov^, *G̅* *^r^* ^,cov^, MVcov (*G̅* ^cov^) and VTcov (*V̅* ^cov^) of the same space *k*(*k*−1)/2 were defined as follows. The element of VTcov vector is thus 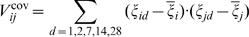, where 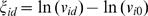, and 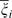 is the average of 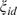 across days 1, 2, 7, 14, and 28. Similarly, for the *r* ^th^ gene, 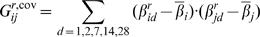, where 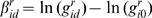 and *β̅* *_i_* is the average of *β^r^_id_* across all genes and all days.

The associations between vectors G, GD and vectors MV and VT both for Euclidian and Covariance metrics were estimated by the Pearson correlation coefficients. We assume that (i) the expression of the gene log-signal in a patient is normally distributed, (ii) expression values of a gene across patients are independent, and (iii) for all genes there are no dependences of between-patient distances according to gene expression and between-patient distances according to viral titer values. The method detects linkage of genes with viral titer as statistically significant deviations against the point (iii), i.e., differences in correlation coefficients from 0. The statistical significance of correlation coefficients may be calculated using Fisher's Z transformation[16] for correlation coefficients which is distributed normally with mean zero and variance 1/[{*k*(*k*−1)/2}−3]. We can alternatively estimate the significance of *R* ^2^ (the square of the correlation coefficient) assuming a beta distribution with 0.5 and *k*(*k*−1)/2 degrees of freedom. If we take into consideration that there is a weak mutual dependence between each pair of patient distances with the same patient is in both pairs, the significance estimation according to beta distribution with 0.5 and *k*(*k*−1)/4 degrees of freedom will compensate this weak mutual dependence of inter-patient distances.

### MV and VT relationship and selection of VT-linked genes

In order to take into account both Euclidean and Covariance measures of proximity in the selection of VT-linked genes, the analysis was done as follows. A multidimensional scaling[17] was applied to reduce the dimensionality of the *k*(*k*−1)/2 space. Namely, the mutual proximities of gene (G) and gene-day (GD) vectors estimated through their correlations with MV and VT vectors (vectors are prepared according to Euclidean and covariance measures) were represented by two-dimensional Euclidean distances in the principal component analysis (PCA) [12] space. Two principal components (PC1 and PC2) were detected according to 8 initial variables, these variables being correlation coefficients for vector pairs (Gcov-MVcov), (GDcov-MVcov), (Gcov-VTcov), (GDcov-VTcov), (Geu-MVeu), (GDeu-MVeu), (Geu-VTeu), (GDeu-VTeu) across all gene-days. The first principal component appears to be defined by correlations of G and GD vectors with MVcov and MVeu vectors, and the second one by correlations of G and GD vectors with VTcov and VTeu vectors. This implies that gene (G and GD) correlations with two MV vectors are independent from gene correlations with two VT vectors, and therefore the general variability of patients according to gene expression is not related to the response to the combination therapy of patients.

The deviation of some GD-vectors from the core of their distribution in the multi-scaling space in the direction of VT vectors (i.e. at PC2 direction) indicates a link of these gene-days (and genes) with viral titers. As the initial criterion for gene-day detection the PC2>threshold was used. The statistical significance of such gene detection was checked by Fisher's Z-test and via beta distribution for correlation coefficients of G(GD) vectors regarding VTcov vector. Both tests used an adjusted number of degrees of freedom to compensate for a weak inter-dependence of vector coordinates.

The more accurate check of the gene detection significance and the estimation of False Discovery Rate (FDR) were performed through permutations. In the first step the distribution of “random” gene-days in the multiscaling space was prepared through permutation of gene-days log-signals over 52 patients of the study. After that correlations of permutated gene-day based inter-patient matrices with VT and MV vectors in k(k−1)/2 space were calculated, and the previously found transformation of the initial 8-dimension space into the multiscaling space was applied to these correlations.

The FDR estimation for the detected set of real genes was done as follows. The sub-space that the gene set is occupied in the multiscaling space was defined as the sphere around the central position of this set. The ratio of the number of permutated gene-days inside the sphere (normalized to the size of real gene set) to the number of the real gene-days inside the sphere is the False Discovery Rate estimation for a sphere of the given radius.

## Results

**Figure 1:**
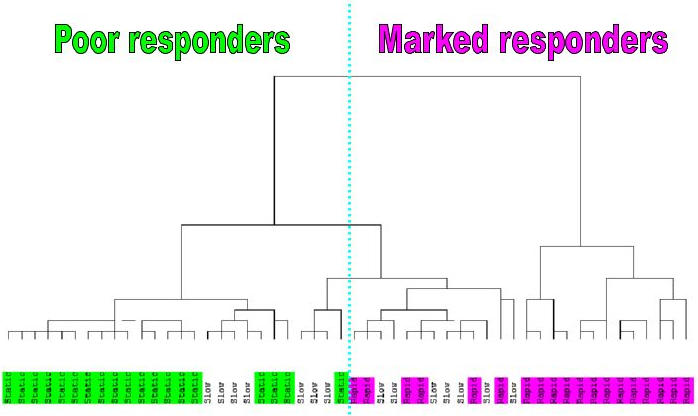
The clustering of 52 patients according to viral titer measurements across days. All viral titer values of each patient are normalized by the same patient day 0 viral titer measurement. The Euclidean measure based hierarchical (UPGM) clustering indicates a good separation of the early response Marked (pink) and Poor response (green) patients. The Slow (yellow) patients are mostly concentrated at the intermediate branch of the clustering tree.

As a first step, the correlation between patient classification based on decrease in virus titer by day 28 and the unsupervised viral titer based clustering of patients was tested. Namely, natural log-transformed viral titers of the 52 patients were normalized using the baseline viral level (i.e. day 0) [i.e. ln(v_i_ )−ln(v_0_) ], where v_i_ is the viral titer value at day i. The clustering of patients was done by the hierarchical UPGMA method [13] using a Euclidean measure of similarity. The branches of the clustering tree (patients) were labeled by type of response according to the initial categorization of the early response (marked, intermediate, and poor) [11] (Fig 1). This hypothesis-free clustering based on inter-patient Euclidean distances according to viral titer demonstrated that patients could be placed into three groups corresponding to the response classification of patients. Indeed, the biggest and most compact cluster consists of poor response patients (the left branch of the tree). The right branch of the cluster contains just marked response patients. The middle branch of the cluster contains a mix of all three classes of patients. If only poor and marked response patients are considered, then there is no misclassification for extreme right and left clusters of the hierarchical classification.

**Figure 2:**
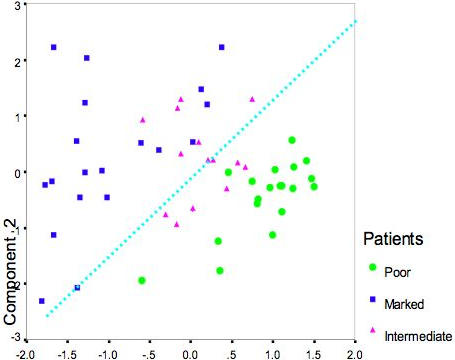
The PCA visualization of inter-patient proximities according to day 0 normalized per day viral titer values. First two Principal Components cover 93% of data variability and thus gives the good presentation of the patient distribution in the 5 dimensional space. There is the clear divergence of Marked (dark blue points) and Poor (green points) response patients. The separation line (dotted blue line) indicates the virtual border between these two populations. Slow patients (pink points) are mostly concentrated along the borderline.

Another compact visualization of patient grouping according to the same baseline-normalized viral titer data was performed using PCA. The first two principal components covered 93% of total data variability. The distribution of patients using the classification given above demonstrated clear separation of poor response patients from marked response patients (Fig 2, see separation line).

**Figure 3:**
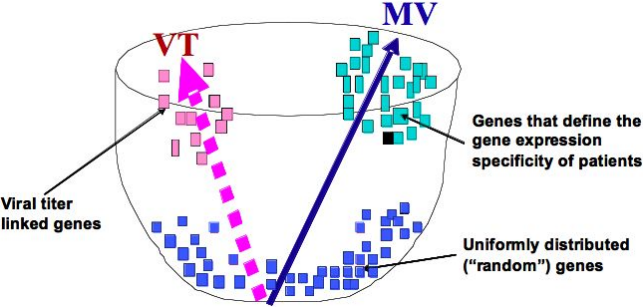
The schematic representation of the algorithm's main idea. Any matrix of inter-patient distances for k patients could be presented as a point (vector) in k (k−1)/2 dimensions space. There are two main vectors in this space; the inter-patient matrix according to viral titer (blue dotted vector) and inter-patient matrix according to expression of all genes (black vector). The expression of every gene is presented as a point (vector) in the same space: the inter-patient matrix according to the expression values of this gene. Some genes could be close to viral-titer vector VT or/and to all gene expression vector MV. Inter-patient matrices according to expression of individual genes (G) at specific days (GD) are dots of the figure. Points of high correlation with VT vector (pink dots) are VT-linked genes. MV linked genes (green dots) define individual variability of patients according to their gene expression. The genes not linked with the two main vectors genes are in red.

**Figure 4:**
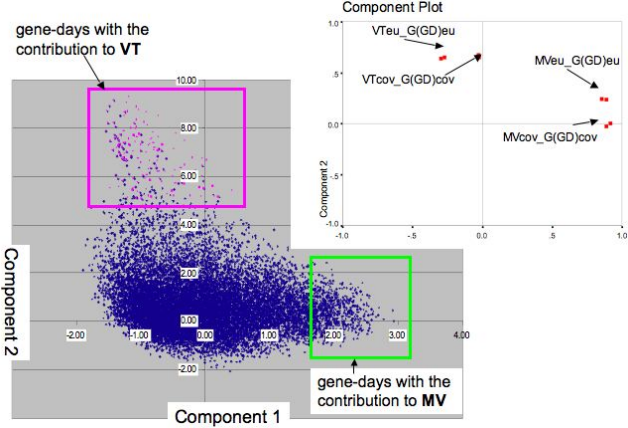
The two-dimensional PCA representation (cover 63% of data variability) of inter-vector correlations in k (k−1)/2 space – the space of distances between inter-patient matrices. One may interpret this figure as the view from above on Fig. 3 schema. Follow to multidimensional-scaling method the proximities between gene based inter-patient matrices as estimated through their correlations with MV and VT matrices were represented by inter-gene (gene-days) Euclidian distances in the PCA two-dimensional space. Positions of VTeu, MVeu (Euclidean metrics) and VTcov, MVcov (covariance metrics) matrices in the PCA space are seen on the insert. VT linked genes are denoted by pink dots. MV-linked genes are in the frame on the figure right.

In the second step, the viral titer linked genes were determined. We defined VT-linked genes as the ones that produced a clustering of patients similar to viral titer based clustering of patients. The procedure for their detection was as follows: Gene expression for each day was normalized with regard to day 0 expression, as was done for viral titer. The determination of genes was based on the hypothesis that the between-patient proximities according to gene and gene-day expression pattern of specific genes are correlated with the viral titer based inter-patient proximities. Thus we looked for genes (gene-days) that “associated” with the viral titer. The selection was done through multidimensional scaling PCA representation for the space of correlations between inter-patient distance matrices (Fig 3). The deviations of some GD-vectors from their uniform distribution in the PCA space in the direction of VT vectors (Fig.4, pink dots) indicated a link of these gene-days (and genes) with viral titer behavior of patients. Thus, gene-days with the second principal component (PC2) value more than 5 were selected. Genes having less than 3 gene-days with PC2 greater than 5 were filtered out.

The correlation coefficients of all detected genes with VTcov (as representing VT group of inter-patient matrices) are more that 0.24 (Table S1) which corresponds to a p-value of less than 1.0E-08 for 1326 (52*51/2) degrees of freedom.

The permutation analysis (see M&M) was based on 1000 permutations of gene-day signals across all 22000 genes at 5 days. The minimal radius of the sphere that covers 80% of gene-days of the selected 37 genes in the 4-mer multi-scaling space is 2.6. This radius corresponds to FDR 1% of the detection. The four dimensional multi-scaling space was taken because such a number of PCA components cover more than 80% of gene expression data variability.

Most of detected genes (Table 1) have been identified previously as interferon induced in PBMC or A549 cells in vitro [7], [18] supporting the conclusion that these are meaningful genes although they were identified in an unsupervised manner. Table S1 lists the top 37 probe sets with details of each probe set and with the day-by-day correlations between gene expression and viral titer.

**Table 1:** Genes hypothesized to be important in the anti-hepatitis C response.

Gene name	Unigene ID	Genbank ID	Gene description
BLZF1	Hs.494326	U79751	Basic leucine zipper nuclear factor.
DDX58	Hs.438386	NM_014314.1	RIG 1 ( helicase)
DNAPTP6	Hs.230767	AK002064.1	DNA polymerase activated protein
EIF3S6IP	Hs.446852	AA862804	Eukaryotic initiation factor 2.
EPHB2	Hs.523329	AI038197	Ephrin B/tyrosine kinase receptor family.
FLJ20035	Hs.481141	AI093428	Helicase
FLJ38348	Hs.546523	AV755522	coiled-coil domain 75 ( CCDC75):RNA binding proteins
G1P2	Hs.458485	NM_005101.1	ISG15 ubiquitin—like modifier.
G1P3	Hs.287721	NM_022873.1	IFI6: inhibitor of apoptosis
HERC5	Hs.26663	AA905126	Ubiquitin ligase 5
HERC6	Hs.435365	NM_017912.1	Ubiquitin ligase
IFI27	Hs.532634	AA991433	Unknown function
IFI44	Hs.82316	BE049439	hepatitis C associated microtubule protein.
IFI44L	Hs.389724	NM_006820.1	histocompatibility 28
IFIH1	Hs.389539	NM_022168.1	Helicase domain 1.
IFIT1	Hs.20315	AA975472	IFI56, Induced protein with tetratricopeptide repeats-1.
IFIT3	Hs.47338	AA991285	Interferon-induced protein with tetratricopeptide repeats 3, ISG60
IFIT5	Hs.252839	N47725	IFI58, induced proteins with tetratricopeptide repeats. 5
IFRG28	Hs.43388	AA970212	Receptor transporter 4
IRF7	Hs.166120	AA991566	Interferon regulatory factor 7
LAMP3	Hs.518448	BX116004	lysosomal associated membrane 3.
MX1	Hs.436836	NM_002462.1	GTP-binding protein
MX2	Hs.926	AI015252	Dynamin and GTPase family
OAS2	Hs.414332	NM_016817.1	2′5′Oligo A synthetase 2, 69/71kD.
OASL	Hs.118633	CB125965	2–5 oligo A synthetase like.
PABPC4	Hs.169900	T05603	Inducible Poly A binding protein
PCTK3	Hs.445402	BC000281.1	PCTAIRE protein kinase 3
PLSCR1	Hs.130759	AI825926	Phopholipid scramblase: enhances anti-viral gene response.
RPL22	Hs.515329	BE250348	60 S ribosomal protein: EB virus binding protein
RSAD2	Hs.17518	AI337069	viperin, cig 2.
SAMD4	Hs.98259	AB028976.1	translation regulator.
SN	Hs.31869	N53555	Sialoadhesion
SNF7DC2	Hs.415534	NM_015961.1	chromatin modifying protein 5 (CHMP5)
TBX3	Hs.129895	NM_006187.1	T-box transcription factor 3.
TRIM5	Hs.350517	AF220028.1	tripartite motif-containing 5
USP18	Hs.38260	AA976038	Ubiquitin specific peptidase

**Figure 5:**
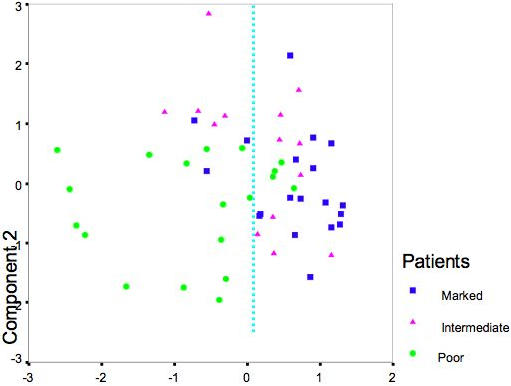
The PCA presentation of the divergence between Rapid and poor response patients according to day 0 normalized expression of 35 VT-linked genes across other days. Two first principal components cover 63% of the data variability. The virtual border line between distributions of Marked and Poor response patients gives three misclassified Marked patients and four misclassified Poor response patients.

**Figure 6:**
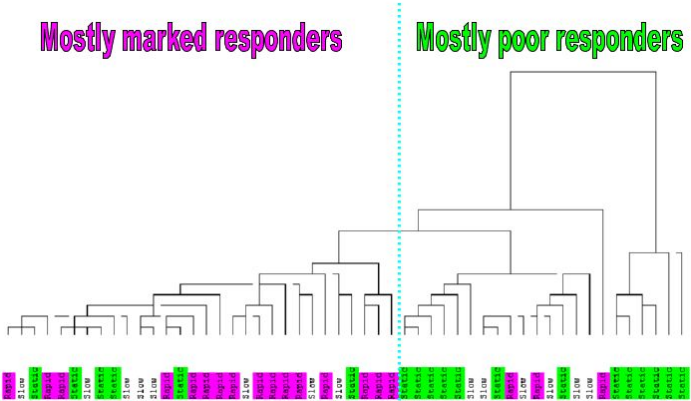
The Euclidean measure based hierarchical (UPGM) clustering of patients according to day 0 normalized expression of 35 VT-linked genes across other days. Marked (pink) and Poor response (green) patients are mostly separated in two branches of the tree. There are three Marked and six Poor response patients misclassified.

Visualization of the sources of variation of patients according to gene expressions of genes- classifiers was simplified through PCA, which reduced the dimensionality of the data into a relatively small number of components. PCA presentation is illustrated in Figure 5. The baseline-normalized expression of these 37 genes (probe sets) appears to be a good discriminator of Marked vs. Poor. Indeed, the PCA presentation of the distribution for 52 patients according to expression profiles across all days of these 37 probe sets demonstrates the obvious shift of poor response patients to negative values of the component 1 (Fig 5). The PCA presentation of the data distribution is relevant because the first two components of PCA analysis cover a major portion (63%) of total data variability. One can see the same basic separation of marked vs. poor response patients in clustering in Fig. 6.

## Discussion

The goal of this paper is to link heterogeneous sets of observations (gene expressions and viral levels) without an a priori hypothesis. We developed a mathematical model that can be applied to any situation using gene expression and viral titer or any other attributes. Application of this approach to patients treated with interferon/ribavirin is based on the assumption that distances between sets of patient's attributes reflects a biological demarcation of patients. As patient attributes we applied the following measurements: (i) viral titer profile during the first four weeks of treatment (viral levels on days 0, 1, 2, 7, 14, and 28), and (ii) the expression of all 22,000 genes of the Affymetrix array across the time course of the treatment.

The difference among patients according to all 22,000 genes on the array reflects the overall gene expression heterogeneity of hepatitis C patients undergoing interferon/ribavirin treatment. This could be due to differences in response, to genetic heterogeneity, or to difference in arrays and handling of RNA. The differences between patients according to any single gene reflect the gene-specific variability of the hepatitis C patients. The gene-specific variability that correlates with the viral titer variability is what is analyzed in this paper and which may be independent of the above. No differences were found in gene expression in an analysis of RNA isolated from PBMC before treatment, when patients were divided into responders and non-responders. Thus the differences are reflection of treatment rather than a reflection of the course of hepatitis C infection.

The gene-specific divergence of patients is checked against the overall patient divergence according to all genes. It appears that the process of virus clearance by interferon/ribavirin is not the major part of the overall gene expression pattern. Indeed, VT-linked genes (genes identified as viral clearance related) make a rather small input into the MV vector, which represents the pattern of overall gene expression variability of patients (Fig.3 and 4).

We examined the variability of patients according to changes in viral titer with time. This analysis demonstrates that the clustering of patients [Fig. 1] according to viral titer largely matches the a priori definition for classification of patients (marked, intermediate, poor) used in this and earlier papers [11]. Although there are a few outliers, in general our initial division using day 28 viral titer appears to have biological as well as mathematical relevance. Even though the a priori classification of patients was based solely on the viral decline at day 28 relative to day 0, the unsupervised clustering of patients based on viral titers at all 6 days resulted in similar clusters, indicating that patterns of viral declines for marked, intermediate and poor response patients are different in-between day 0 and day 28. Since the clustering of patients according to the viral titer profiles is in good correspondence with patient classification (Fig. 1,) the selected 37 probe sets (table 1 and table S1) could be considered as genes that are important in the anti-viral and immune response to interferon/ribavirin therapy.

A very large number of genes are modified by the treatment *in vitro* of PBMC with interferon and ribavirin [7], [19]. Similar results have been found recently following the treatment of A549 cells and Huh 7 cells with IFN-αcon1 [10]. Many of the genes listed in table 1 are known to be involved in the interferon response from *in vitro* studies [7], [10], [18]. In most cases the level of induction for these genes was much lower in poor response patients than in marked patients, as would be expected from the relationship to viral titer.

Among the genes we identified as important is IRF-7. This gene is required for the induction of type I interferons [20]–[23] and is an important component of the Toll signaling pathway [24]. Studies using LPS have indicated that IRF-7 plays an important role in LPS induced B7.1 activation through the JNK system [25]. B7-1 is an important co-stimulatory factor required for T-cell activation. Binding of TLR7 and activation of the NFkB pathway via IRF-7 [26] is required for the endogenous production of interferon-alpha and beta. Thus the levels of IRF-7 may be important in the overall patient response [23], and the endogenous production of IFN-α or IFN-β would certainly lead to further anti-viral effects. Another gene recently reported to be involved in the signaling of the interferon response, is RIG-I, a key ds-RNA sensor protein. Activation of RIG-I (DDX58) leads to the induction of IRF-3, IRF-7, and NFκB [27]–[29]. This pathway is blocked in vitro in hepatitis C infection by the NS3/4A protein of the virus [29]. However it is unlikely that this occurs in PBMC, since there is no evidence that PBMC are widely infected with the virus. IFIH1 is also a DEAD protein with a helicase domain. Its relationship to RIG-I is unknown. Among the other genes listed in Table 1, many have been describes as part of the initial response in vivo (in Chimpanzees) to hepatitis C infection, and are the results of endogenous interferon production. These include cig 5, (RSAD2), IFI44, MX1, and the OAS genes [30]. Viperin (cig5) has also been reported as being induced in the liver in human patients during hepatitis C infection, presumably by endogenous interferon or double-stranded RNA [31]. It has been proposed that this gene product has anti –hepatitis C activity [31]. PLSCR1 has also been shown to potentiate the anti-viral activity of interferon . When PLSCR1 expression was decreased by siRNA, higher titers of VSV and EMC were obtained [32]. G1P3 (IFI-16-6) may function as a cell survival protein by inhibiting mitochondrial-mediated apoptosis through the inhibition of caspase 3 activity [33]. Its role in the anti-viral response or anti-hepatitis response is unknown. .. Many of the genes on this list have unknown functions (IFI27, IFIT1, IFIT5, , IFI44, IFI44L, LAMP 3, , FLJ20035, IFRG28, DNAPTP6). Other genes are involved in transcription regulation including RGL1, and TBX3 or translation repression (SAMD4).). PABPC4 is also known as inducible poly(A) binding protein and is upregulated in activated T-cells, but not in resting T-cells [34]. However in this study it was slightly down regulated. It also appears to be involved in the regulation of IL-2 [35]. G1P2 is identical to ISG15. This protein is a ubiquitin-like protein which is conjugated to many cellular proteins [36]. It appears to interfere with protein poly-ubiquitination and protein degradation. Its role in the interferon response is unknown, although it is highly induced. USP18, a member of the de-ubiquitinating protease family of enzymes, removes ubiquitin adducts from a broad range of protein substrates. Herc 5 and Herc 6 belong to a family of ubiquitin ligases. Thus many genes involved in protein modification are essential to the anti-viral response. SN (Sialoadhesion) very highly induced both in vitro and in vivo is a member of the sialic acid binding immunoglobulin ( Ig)-like lectins. And is primarily expressed in resident and inflammatory macrophage populations [37], [38]. SN deficient mice exhibit changes in B and T-cell populations and it is proposed that this molecule regulates the immune system [39].

Two genes that are down regulated correlate with the viral response: RPL22 (ribosomal protein L22), a component of the 60S ribosome, and eukaryotic translation initiation factor 3, subunit 6 interacting protein. Whether this decrease is involved in virus inhibition through modifying IRES-dependent translation of the HCV genome is speculative.

It is of interest that not only inducible genes appear to be a major component of the interferon response, but also down regulation (repression) of a translation factor and ribosomal protein.

Since this was an unsupervised analysis and did not take into account A/P ( absence/present) filtering, some of the genes are possibly not involved in the anti-viral response, since they were not present in specific classes of patients as analysed with MAS5 soft ware. These include BLZF1, EPHB2, PCTK3 and SNF7DC2

In summary we have identified key genes in the response to interferon/ribavirin in hepatitis C patients using a novel method of analysis. This is based on correlation with decrease in virus titer. This method has broad utility and can be used to analyze response to any group of factors influencing biological outcome.

## Supporting Information

Table S1 Contains 166 gene-days of 37 viral titer linked genes (probe sets). Not less than 3 gene-days of the gene have component PC2 values more than 5 (these gene-days are pink dots of the Fig. 4). The component PC2 is PCA second component after multidimensional scaling of 8 variables: correlation coefficients in the space of (52*51)/2 dimensions between Gcov_MVcov, GDcov_MVcov, Gcov_VTcov, GDcov_VTcov, Geu_MVeu, GDeu_MVeu, Geu_VTeu, GDeu_VTeu for all gene-days. Columns of the Table S1 are as follows: SID - Affymetrix probe set ID; DAY - day of the treatment; Static_CA_1013 - log-transformed and day0 normalized gene expression values [(Ln(d_i_)- Ln(d_0_))] for a patient 1013. The response based classification of the patient and her/his race are identified in the patient's name; Slow_CA_1016; Rapid_AA_1018; .................. - 52 patients of the study; VTeu_Geu - the following 8 columns are correlations between corresponding vectors in the space of (52*51)/2 dimensions. These correlations are 8 initial variables for the multidimensional scaling procedure; VTeu_GDeu; MVeu_Geu; MVeu_GDeu; VTcov_Gcov; VTcov_GDcov; MVcov_Gcov; MVcov_GDcov; GENE_SYMBOL - the following are gene names and descriptions; LOCUSLINK; UNIGENE; GENBANK; GENE; SOURCE_ID; DESCRIPTION; PC1 - the multidimensional scaling first component; PC2 - the multidimensional scaling second component.
(0.23 MB XLS)
